# Integrating Artificial Intelligence and PET Imaging for Drug Discovery: A Paradigm Shift in Immunotherapy

**DOI:** 10.3390/ph17020210

**Published:** 2024-02-06

**Authors:** Jeremy P. McGale, Harrison J. Howell, Arnaud Beddok, Mickael Tordjman, Roger Sun, Delphine Chen, Anna M. Wu, Tarek Assi, Samy Ammari, Laurent Dercle

**Affiliations:** 1 Department of Radiology, New York-Presbyterian Hospital, Columbia University Vagelos College of Physicians and Surgeons, New York, NY 10032, USAhjh2136@cumc.columbia.edu (H.J.H.); 2 Department of Radiation Oncology, Institut Godinot, 51100 Reims, France; 3 Department of Radiology, Hôtel Dieu Hospital, APHP, 75014 Paris, France; 4 Department of Radiation Oncology, Gustave Roussy, 94800 Villejuif, France; 5 Department of Molecular Imaging and Therapy, Fred Hutchinson Cancer Center, Seattle, WA 98109, USA; 6 Department of Radiology, University of Washington, Seattle, WA 98195, USA; 7 Department of Immunology and Theranostics, Beckman Research Institute of City of Hope, Duarte, CA 91010, USA; awu@coh.org; 8 International Department, Gustave Roussy Cancer Campus, 94805 Villejuif, France; 9 Department of Medical Imaging, BIOMAPS, UMR1281 INSERM, CEA, CNRS, Gustave Roussy, Université Paris-Saclay, 94800 Villejuif, France; 10 ELSAN Department of Radiology, Institut de Cancérologie Paris Nord, 95200 Sarcelles, France

**Keywords:** artificial intelligence, radiomics, PET, PET/CT, immunotherapy, drug discovery

## Abstract

The integration of artificial intelligence (AI) and positron emission tomography (PET) imaging has the potential to become a powerful tool in drug discovery. This review aims to provide an overview of the current state of research and highlight the potential for this alliance to advance pharmaceutical innovation by accelerating the development and deployment of novel therapeutics. We previously performed a scoping review of three databases (Embase, MEDLINE, and CENTRAL), identifying 87 studies published between 2018 and 2022 relevant to medical imaging (e.g., CT, PET, MRI), immunotherapy, artificial intelligence, and radiomics. Herein, we reexamine the previously identified studies, performing a subgroup analysis on articles specifically utilizing AI and PET imaging for drug discovery purposes in immunotherapy-treated oncology patients. Of the 87 original studies identified, 15 met our updated search criteria. In these studies, radiomics features were primarily extracted from PET/CT images in combination (*n* = 9, 60.0%) rather than PET imaging alone (*n* = 6, 40.0%), and patient cohorts were mostly recruited retrospectively and from single institutions (*n* = 10, 66.7%). AI models were used primarily for prognostication (*n* = 6, 40.0%) or for assisting in tumor phenotyping (*n* = 4, 26.7%). About half of the studies stress-tested their models using validation sets (*n* = 4, 26.7%) or both validation sets and test sets (*n* = 4, 26.7%), while the remaining six studies (40.0%) either performed no validation at all or used less stringent methods such as cross-validation on the training set. Overall, the integration of AI and PET imaging represents a paradigm shift in drug discovery, offering new avenues for more efficient development of therapeutics. By leveraging AI algorithms and PET imaging analysis, researchers could gain deeper insights into disease mechanisms, identify new drug targets, or optimize treatment regimens. However, further research is needed to validate these findings and address challenges such as data standardization and algorithm robustness.

## 1. Introduction

The discovery, development, and clinical application of immunotherapy have radically impacted therapeutic approaches to numerous cancers, resulting in a significant impact on survival outcomes for many patients. By exploiting a number of innate biological mechanisms, including regulation of cytotoxic T-cells via checkpoint molecules (e.g., PD-1, PD-L1, CTLA-4, and LAG-3), specifically directed oncolytic viruses, and engineered immunocytokines and T-cells, clinicians have been able to divert from the traditional but highly morbid approaches of chemotherapy and radiation. These treatments are inherently more selective for cancer cells, and they seek to target and overcome the mechanisms by which tumors can evade a patient’s natural defense mechanisms. However, the development of novel immunotherapies is an incredibly intricate process, impeded by challenges not only at the bench-top during target identification and molecular development, but also once a drug has left the lab and entered human trials. Clinical deployment of new immunotherapies is complicated by atypical response patterns previously unseen with chemotherapy, including pseudoprogression, in which a tumor initially appears to grow or spread before eventually regressing, as well as various treatment-related inflammatory conditions that can appear in nearly any organ system. Additionally, only a subset of patients exhibit partial or complete responses with immunotherapy, emphasizing the need for better a priori candidate selection. 

Positron emission tomography (PET), an imaging modality that provides information about tissue metabolic function, is already widely used for initial cancer diagnosis and staging. Further, the combination of PET and artificial intelligence (AI), which provides high-throughput computation and specifically trained pattern recognition, may help improve efficiency at each step of the development pipeline, ultimately allowing for quicker evaluation and implementation of effective therapeutics in patient care.

This review aims to highlight current studies examining the combination of PET and AI for use in patients treated with immunotherapy. We first provide general background information on immunotherapy, as well as its therapeutic counterpart, targeted molecular therapy, in addition to describing the basics of PET imaging and AI. We then discuss the results of our subgroup analysis with a focus on pain points in the drug development process, including target validation, safety, biomarkers for prognostication and response evaluation, and generalizability to clinical application. For each topic area, we describe current challenges and relevant AI/PET studies from our focused review, as well as other related and current advances. We conclude by describing promising developments that could incite future research and impact the field moving forward.

## 2. Background

### 2.1. Targeted Molecular Therapy

While traditional cytotoxic chemotherapy is non-selective and affects both neoplastic and normal host cells, targeted molecular therapies were developed with the goal of improving treatment specificity. This type of therapy generally involves small molecules designed to target mutated proteins or biochemical pathways that allow for unregulated growth and development in tumor cells. The first example in this class was imatinib, a BCR-ABL tyrosine kinase inhibitor found to induce high response rates in chronic myelogenous leukemia [1]. Additional tyrosine kinase inhibitors have since become mainstays of non-small cell lung cancer (NSCLC) treatment, including erlotinib for patients with epidermal growth factor receptor (EGFR) mutations and crizotinib for those with anaplastic lymphoma kinase-positive (ALK+) disease [2]. The former is now part of first-line treatment protocols for NSCLC, and the latter was shown to result in superior survival over first- and second-line cytotoxic chemotherapy regimens [3]. Countless other molecular targets, such as KRAS, BRAF, KIT, HER2, CDK 4/6, and PARP, have been approached with small molecules, but while improved selectivity overall results in fewer off-target effects, these treatments are highly susceptible to resistance through de novo mutations at the target moiety of interest [4,5,6]. 

### 2.2. Immunotherapy

Immunotherapy represents an alternative approach for blunting the tumorigenic properties of cancer and addressing the aforementioned mechanisms of targeted therapy resistance. This type of treatment focuses on immune pathways, inhibiting tumor-mediated processes for immune downregulation while simultaneously amplifying host response against malignancy. As a result, modern approaches to immunotherapy development greatly diverge from the traditional methods of chemotherapeutic drug discovery and are more similar to those employed for targeted therapy. When creating a novel immunotherapy, researchers first identify a suitable or theoretically relevant target via cell lines, animal models, or bioinformatics before subsequently exploiting cellular machinery to develop antibodies highly specific for proteins expressed on cancer cells. As an example, ipilimumab, one of the earliest FDA-approved immunotherapies, was developed after the identification of the CTLA-4 cell surface receptor, a protein that functions as an immune checkpoint inhibitor (ICI), suppressing host immune defenses [7]. In theory, blocking this receptor on cancer cells would reduce inhibition of the host immune response, leading to the destruction of malignant cells. By utilizing a transgenic mouse model and capitalizing on the natural processes of class-switching and somatic mutation, researchers were able to generate a massive library of IgG antibodies, subsequently identifying those that were most selective for CTLA-4 [8]. The isolated antibodies were then validated in both in vitro and in vivo mouse models to ensure selectivity and efficacy against tumors.

It is important to note that combinations of immunotherapy with cytotoxic chemotherapy or small molecule inhibitors may beget even more significant responses, and tandem therapy remains an active area of research [9,10].

### 2.3. PET Imaging

Positron emission tomography (PET) is a medical imaging technique that quantifies the distribution of radioactive molecules throughout the human body. The most commonly employed radiotracer, ^18^F-FDG, is composed of a radioactive fluorine atom paired with a glucose moiety, allowing clinicians to visualize in vivo glucose metabolism. For years, ^18^F-FDG PET imaging has been the standard of care in oncology for the initial assessment and staging of cancerous lesions. PET has also been employed in response assessment, allowing clinicians to track the regression, malignant spread, and recurrence of cancer by analyzing changes in metabolic activity over time [11]. Recently, the scope of PET has widened in light of new developments in radiotracer technology, generally involving the linkage of radiolabels and highly targeted monoclonal antibodies. PET imaging is primed to play a role in future oncologic drug development due to its current status as the gold standard for diagnosis and staging, wide availability, and ease of technical modification and development.

### 2.4. AI and Radiomics

Fundamentally, medical images are made up of two-dimensional pixels (or three-dimensional voxels) that each have a corresponding brightness (or intensity/gray-level) value. The amalgamation of many different individual pixels of varying brightness values creates a macroscopic image from which a radiologist generates an interpretation. However, there is an immense amount of potentially valuable data buried in the micro-scale relationships between individual pixels that may hold clues to underlying biological processes imperceptible to the human eye. Additionally, these relationships cannot be reproducibly quantified by clinicians (due to inter- and intra-observer variability) and are difficult to compare and combine into complex decision algorithms. Artificial intelligence, and in particular the subcategory of radiomics (i.e., extracting textural information from medical imaging through mathematical processes), could be used to address these challenges by quantifying relationships between pixel intensities through discrete imaging “features”. These features describe patterns such as the maximum, mean, and distribution of brightness values in a certain image, as well as symmetry, heterogeneity, and geometric co-occurrences (i.e., spatial relationships of all pixels with identical intensities) of pixels in a region of interest. In theory, by using radiomics and a large dataset of medical images for model training, researchers could identify a precise combination of imaging features and relative weights to create an “imaging phenotype” that is correlated to a particular disease process or patient outcome. Model outputs are generally binary, classifying patients as likely or not likely to have a particular outcome, but even this information can be essential to progressing care or facilitating clinical trials.

## 3. AI and Immunotherapy: Current Landscape

A 2022 scoping review surveyed three databases, MEDLINE (PubMed), CENTRAL (Cochrane Central Register of Controlled Trials), and EMBASE, for all studies through 26 February 2022, using search terms related to artificial intelligence and radiomics applied to the medical imaging of immunotherapy-treated cancer patients [12]. There was one additional relevant study added that was identified in references but did not appear in the original search. Studies were included if they involved the immunotherapeutic treatment of human cancers or models of human cancers and if they employed radiomics with PET, CT, or MRI imaging. Case reports, systematic reviews or meta-analyses, perspectives, editorials, book chapters, workshop reports, conference abstracts, and non-English language reports were excluded. In total, 87 studies were recorded.

**Figure 1:**
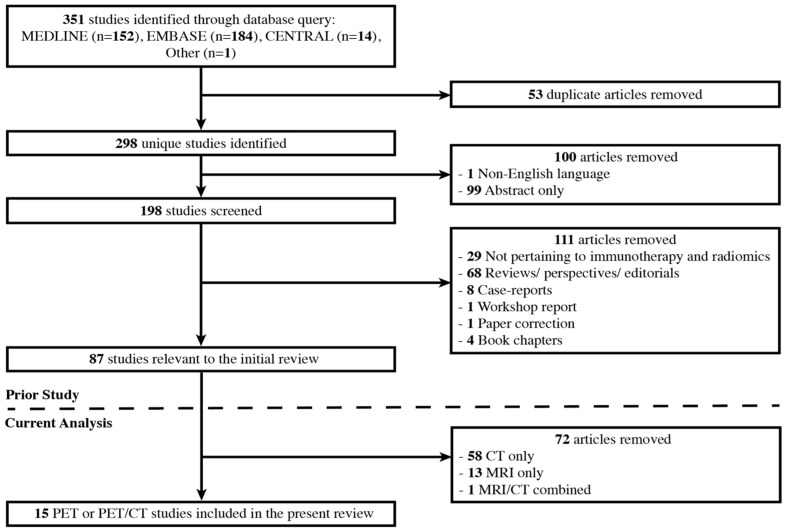
Study selection process for the original 2022 review and the present PET and PET/CT second-look analysis.

To survey the current state of artificial intelligence specifically applied to PET imaging, we performed a focused second-look analysis of this review, examining studies that involved the extraction of imaging features from ^18^F-FDG PET and CT images in combination (9 of the 87 total studies, 10.3%) and those utilizing only PET images (*n* = 6, 6.9%). The complete study selection process, including that of the original review, is displayed in Figure 1.

**Figure 2:**
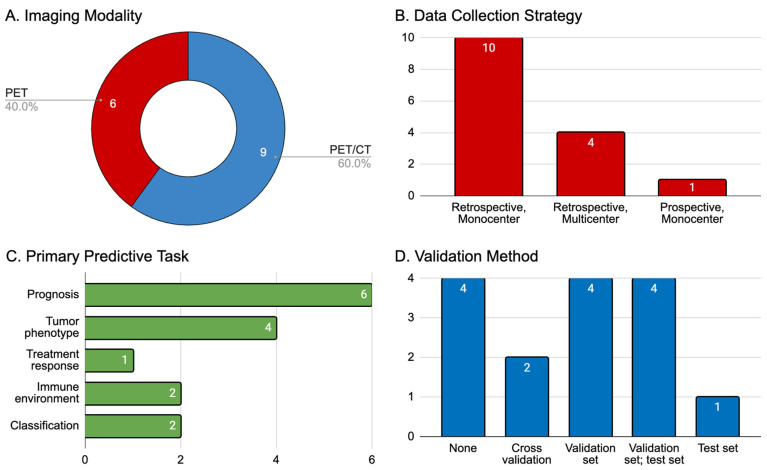
Summary of 15 articles that met our inclusion criteria. (**A**) Imaging modality employed (PET indicates ^18^F-FDG-PET imaging, CT indicates computed tomography); (**B**) data collection strategy classified by time course (retrospective or prospective) and center involvement for patient recruitment (single or multiple institutions); (**C**) primary predictive aim of the AI model trained in the study: prognosis (e.g., measures of overall or progression-free survival, or durable clinical benefit), tumor phenotype (e.g., PD-L1 receptor expression), treatment response (e.g., predictions of clinical endpoints as defined by RECIST 1.1 or similar criteria), tumor immune microenvironment (e.g., tumoral infiltration of CD8+ T-cells); (**D**) validation method used for AI models.

We explored this subsection of 15 articles for the cancer of interest, the primary (and if applicable, secondary) predictive task of the radiomics model (e.g., prognosis, treatment response, tumor phenotyping, etc.), the data collection strategy employed (prospective or retrospective and single- or multi-center), and the validation method used to stress-test each radiomics model. The primary cancers of interest were NSCLC (*n* = 11, 73.3%), melanoma (*n* = 3, 20.0%), and lymphoma (diffuse-large B-cell lymphoma, *n* = 1, 6.7%), with the former two diseases also representing the most common indications for immunotherapy overall. The median [interquartile range] size of aggregated patient cohorts was 132 [148], with the largest number of patients (*n* = 697) having been recruited in a study of NSCLC. Summaries of these articles are displayed in Table 1 and Figure 2.

## 4. Target Validation

Medical imaging and AI can help researchers and clinicians identify the presence and distribution of specific molecular targets, aiding in the deployment of patient-tailored treatments.

### 4.1. Challenges

Immunotherapy works by targeting very specific mechanisms exploited by tumor biology to either suppress or evade the host immune system. Characteristics of individual tumors, such as PD-L1 and CTLA-4 expression, as well as tumor immune cell infiltration, indicate that a patient’s specific disease may be particularly susceptible to certain types of treatment. The current standard for molecular assessment involves using immunohistochemistry to analyze a tissue sample collected via biopsy. Acquiring these samples is invasive and not without risk, leading clinicians to minimize their use of the procedure, typically only performed before treatment initiation or at disease relapse. Biopsies are also limited in both time and space, leading to a chance of mischaracterizing disease. Additionally, tumors are innately heterogeneous, with different cells possessing varying combinations of mutations, allowing for evolution as treatment is applied [28,29]. Cells with more susceptible phenotypes will die, while those less specifically targeted by a treatment will live on, changing the distribution of molecular expression within a tumor over time. Lastly, even at a single time point, a simple biopsy may fail to capture all cell phenotypes represented within a tumor and would not account for inter-tumoral heterogeneity if only the primary tumor was sampled [29].

### 4.2. State of the Art

In the 15 original studies reviewed, AI was used for classification of either tumor phenotype (*n* = 4, 26.7%), primarily in describing PD-1/PD-L1 expression, or tumor immune microenvironment (TME, e.g., tumor infiltration by host immune cells, *n* = 2, 13.3%). The former studies built radiomics models using medical imaging for prediction of PD-L1 overexpression in a certain percentage of cells, i.e., a model would generate a binary output that roughly translated to a prediction that over or under 1% of cells within an analyzed tumor had increased expression of PD-L1. Importantly, these models were built using radiomics features extracted from the primary tumor alone and correlated to known PD-L1 expression confirmed by histologic analysis, which remains the current ground truth for comparison. In describing TME, studies sought to quantify the infiltration and distribution of CD8+ T-cells within a tumor, as well as describe immune cell transcriptomic signatures as a proxy for immune activity.

These classifications correlate with a tumor’s likelihood of treatment response—a tumor with high PD-L1 expression or increased infiltration by host immune cells is more likely to respond to immune-activating or checkpoint-inhibitor therapy. In clinical trials, quantifying tumor character a priori may allow for more directed recruitment of patients, facilitating the identification of populations for which the efficacy of novel therapeutics is likely to be high. In other words, response rates of a PD-L1-inhibiting monoclonal antibody will be much higher in a cohort of highly PD-L1-expressing patients than in a mixed cohort with unknown PD-L1 expression, overall increasing the effect size and lowering the recruitment requirements necessary for a positive trial.

An additional approach aimed at resolving the time scale limitations of traditional tissue biopsies is the evaluation of free circulating tumor DNA (ctDNA), so-called “liquid biopsies.” ctDNA is a subdivision of circulating free DNA (cfDNA, or genetic material shed into general circulation by any cell, especially those undergoing apoptosis) that is specific to cancer. Detecting these ctDNA fragments in patient plasma can allow for non-invasive time-dependent monitoring of tumor genetic profiles, responses to treatment, and metastases, among many other metrics. A full discussion of ctDNA application is beyond the scope of this review, but has been covered extensively by other groups [30,31,32,33,34].

## 5. Safety

Imaging can help clinicians evaluate immunotherapy-induced toxicity and perform risk stratification, allowing for careful decision making on whether or not to proceed with further treatment.

### 5.1. Challenges

Establishing standard dosing regimens for new immunotherapeutic agents remains challenging. Insufficient dosing may fail to stimulate a patient’s native immune system, resulting in little or no clinical effect, while supratherapeutic doses may precipitate side effects severe enough to dramatically increase morbidity and mortality or lead to treatment interruption [35]. By definition, some classes of immunotherapy, specifically ICI, release the “brakes” of a patient’s immune system, often resulting in the collateral damage of native tissues and the induction of a number of inflammatory conditions such as pneumonitis, colitis, hepatitis, and pancreatitis [36]. These side effects are known as immune-related adverse events (irAEs) and are feared complications in immunotherapy due to their ability to derail the application of treatment. irAEs are also difficult to track as they may be delayed compared to the adverse events associated with more traditional therapies, potentially necessitating longer trial durations [37]. Lastly, there is an intricate differential for in-treatment inflammatory changes on imaging (e.g., pneumonitis must be differentiated from cancer progression or radiation therapy toxicity), which must be fully explored in order to make the most appropriate judgment on ongoing treatment plans.

### 5.2. State of the Art

Prediction of sequelae from treatment (e.g., irAEs) was facilitated by AI in 2 studies (13.3%). Both studies utilized pretreatment imaging; one predicted the development of colitis, pneumonitis, Guillain–Barré syndrome, hepatitis, myalgia, or rash, while the other predicted the development of cancer-related cachexia in patients treated with ICI. Both studies utilized weighted multivariate logistic regression analysis to build models correlating radiomics features to specific adverse events, and one of the studies validated its model on a prospectively collected cohort of patients. Even without AI, some groups have previously demonstrated that PET imaging has the capacity to capture 75% of irAEs [38,39].

Identification of adverse events at the earliest possible moment, or prediction of which patients will likely experience adverse events due to innate, baseline physiology, is crucial to avoid delaying the administration of life-prolonging treatment. Having an accurate read of irAE risk would improve clinical agility in switching patients between therapeutic regimens. Additionally, ending negative trials, or those in which patients are at risk for high-morbidity adverse events, would promote patient safety. This could reduce barriers to patient enrollment and improve trial retention, while also helping to address disparities in communities traditionally underrepresented in clinical trials.

## 6. Biomarkers for Prognostication and Response Evaluation

Artificial intelligence allows for the creation of patient-specific imaging “phenotypes”, which can be analyzed before, during, or after treatment. In this model, textural features extracted from images act as biomarkers for the patient’s disease, allowing clinicians to not only prognosticate by using large patient populations with similar phenotypes and known outcomes, but also monitor changes in the imaging manifestations of underlying biological processes during treatment.

### 6.1. Challenges

There is a high bar for the approval of new drugs entering clinical trials; new therapies must demonstrate clear evidence of clinical benefit, defined as an improvement in survival, quality of life, or physical functioning. Beyond this, additional endpoints that have been used in accelerated approvals include the demonstration of more rapid tumor size reduction or longer delay in growth than with traditional therapy, as well as improved progression-free survival (PFS) or complete response (CR) in the absence of treatment-related side effects. Trials are designed around achieving these goals, and any tools that can facilitate this process are invaluable in accelerating approval or preventing setbacks.

A factor complicating clear demonstrations of benefit is that traditional methods of monitoring disease response to treatment using medical imaging (e.g., RECIST, RANO, Cheson, PERCIST, among others) have proven insufficient in capturing the novel tumor behavior seen with immunotherapy. This includes pseudoprogression (initial apparent growth of a tumor before eventual response), mixed responses (certain lesions respond more than others), and hyperprogression (accelerated growth after treatment administration) [40,41,42]. Pseudoprogression may lead to early termination of treatment due to perceived progression, while a true mixed response might compel clinicians to continue treating with the same regimen instead of appropriately switching therapy to address intrinsic tumor heterogeneity. Hyperprogression is far more sinister and may lead to disastrous outcomes from rapid disease progression of unknown origin if not recognized at the earliest possible moment [43].

### 6.2. State of the Art

Per our review, AI was most commonly employed for prognostication (*n* = 6, 40%), either for predicting overall survival (OS) at a specific time point, progression-free survival (PFS, i.e., time from onset of treatment until disease progression or relapse), or durable clinical benefit (DCB, i.e., progression-free survival past a certain predefined time point). One study utilized AI to make baseline predictions about treatment response compared with eventual PERCIST outcomes. No studies specifically examined the differential classification of atypical response patterns discussed above, and thus this remains an area of potential exploration.

At baseline, evaluating the quality of a patient’s tumor and utilizing imaging to predict the likelihood and estimated time frame of survival is crucial for informed decision-making and the selection of a proper course of treatment. More hostile tumors, signified by a lower estimated OS, require more aggressive therapeutic strategies, influencing the treatment outcomes of clinical trials they are involved in. Trial length, an important factor for cost and time considerations in drug development, could also be shortened via early termination if patients displayed imaging phenotypes indicating a low probability of a good outcome with the continuation of a particular treatment. Furthermore, saving money by avoiding failed trials could open the door for the evaluation of additional new drugs, including those for orphan conditions.

In addition to the biomarkers mentioned above, a modified version of the RECIST consensus guidelines has been developed, termed iRECIST, with the intent of addressing atypical tumor behavior observed in response to immunotherapy [44]. A major improvement of iRECIST is its ability to more precisely discriminate instances of progression by including definitions for unconfirmed and confirmed progressive disease (iUPD and iCPD, respectively). When an increase in size or novel lesions are first observed on imaging after treatment initiation, the patient is noted to have iUPD until subsequent assessment, at which time persistence of growth or spread would indicate iCPD. Importantly, patients with iUPD that eventually regresses are eligible for partial response, complete response, or stable disease designations, whereas in standard RECIST, any degree of observed progression precludes attainment of these statuses.

## 7. Challenges to Clinical Application

As discussed, medical imaging helps clinicians monitor the therapeutic effects of a drug and can help guide decision-making during different stages of clinical testing and implementation. However, in order for AI to be applied in more widespread clinical settings, extensive validation must be performed to ensure that simulated performance will be maintained in new populations.

### 7.1. Generalizability

While individual studies may report impressive performances for AI models in isolation, the interpretation of these results must be carried out with caution. The applicability and generalizability of models beyond the initial study populations depend on the quality of patient cohorts used for model training and the rigor of subsequent model validation. Per our review, nearly all studies (*n* = 14, 93.3%) recruited patients retrospectively, and most were based at single institutions (*n* = 11, 73.3%). One study included a prospective testing cohort for model validation. In general, training predictive algorithms on patients from multiple institutions allows the model to be exposed to more heterogeneity between patients and imaging acquisition techniques. Additionally, collecting data prospectively helps to eliminate the bias of retrospective analysis, further supporting the internal and external validity of results.

Model validation is a crucial step in AI development and represents the first real test of predictive ability: applying an algorithm to data separate from what was used for training. Validation methodology can be graded based on the quality of the cohorts used, with the lowest quality validation cohort involving reuse and reorganization of the training set, known as cross-validation. The use of a validation set involves splitting recruited patients into two buckets, one for model training and the other for validation. Alternatively, a validation set can be defined as a cohort of patients recruited separately from the training set, but at the same institution using similar inclusion criteria. In this way, and regardless of the approach, both training and validation sets will have similar patient distributions and characteristics, but the model is being tested on patients that it has not previously been exposed to. A more rigorous validation method involves testing models using a validation set immediately followed by a test set (denoted by us as “validation set; test set”), with the latter defined as a cohort of patients independent from the training/validation sets (i.e., patients were recruited from a different database/institution, recruited using different criteria, or received a different treatment). This strategy provides the highest external validity for AI, as model performances are observed in truly independent cohorts, indicating potential for broad application. Test sets, as defined above, could also be used in isolation, independent of validation sets. Per our review, most studies used validation strategies involving either a “validation set; test set” (*n* = 4, 26.7%) or a “validation set” alone (*n* = 4, 26.7%). Surprisingly, four (26.7%) studies reported no validation whatsoever, while two (13.3%) reported only cross-validation on the training set. One study used a test set in isolation. The relative absence of substantive validation is a trend observed previously in the field, with a 2019 meta-analysis finding that only 6% of studies surveyed in a pool of 516 performed any sort of external validation, while none validated radiomics models with prospective, multicenter external cohorts [45].

### 7.2. Radiomics Quality Score

While scrutinizing the rigor of validation allows for some ability to judge the quality of an AI model, there is a need for a more standardized approach to evaluation in order to properly recognize the relevance, predictive ability, and potential for application of each individual algorithm. Lambin et al. proposed a 16-point scoring system that accounts for, among other metrics, thoroughly described research methods, quality of validation, and description of biological correlates [46]. Although this measure, called the radiomics quality score (RQS), does not take into account sample size or discrete model performance, it allows for standardized assessment and comparison of AI studies. Overall, higher scores (with a maximum score of 36) indicate higher quality studies that can be more easily translated into practice. Of the 15 studies reviewed, the median [interquartile range] radiomics quality score was 15 [10], and the highest score achieved was 24.

## 8. Future Directions: PET-Specific Radiotracers

An emerging technique, immunoPET, seeks to circumvent the use of imaging feature proxies for biological phenomena by more directly interrogating tumor biology through radiotracers appended to monoclonal antibodies (mAbs). The primary molecular targets of these highly specific mAbs are markers on CD8+ T-cells, as well as PD-L1 proteins. Cancers with low levels of CD8+ T-cell infiltration, indicating a lack of immune access to a tumor, or high PD-L1 expression, signaling a blunting of the host immune response, are less likely to respond to immune mobilization via immunotherapy. As previously discussed, a precise description of these variables, based on the pattern and intensity of radiotracer uptake as measured by PET, would allow for a curated selection of patients for trials, identifying those most likely to achieve durable clinical benefit.

Building on a decade of in vivo experimental studies using targeted radiotracers, recent proof-of-concept and Phase I trials have deployed a radiotracer comprised of an ^89^Zr atom tethered to a minibody (engineered antibody) specific for human CD8 protein to evaluate whole-body cytotoxic T-cell distribution and tumor infiltration [47,48,49,50]. Additionally, and perhaps more widely investigated at present, are agents targeting the PD-L1 co-receptor. Radiotracers built from ^64^Cu-, ^89^Zr-, ^18^F-, ^111^In-, and ^99m^Tc-containing scaffolds with high-affinity PD-L1-specific peptides are being used in pre-clinical studies to precisely define tumor surface protein expression [51,52,53,54,55]. Human studies are also underway, with a recent trial of 22 patients demonstrating that PD-L1-specific ^89^Zr PET tracer uptake was more strongly correlated with outcomes than immunohistochemistry or RNA sequencing-based biomarkers [56]. Another trial used an ^18^F-labeled anti-PD-L1 Adnectin protein and demonstrated higher tracer uptake in treatment-responsive NSCLC [57]. A potential future avenue of investigation involves using radiomics or AI not as surrogate measurements for tumor microenvironment, as was performed in many of the studies described in this literature survey, but instead as a means to more thoroughly describe the behavior of these highly specific radiotracers. AI could be used to quantify patterns of radiotracer uptake and distribution that can be compared inter- or intra-patient in a longitudinal series, ultimately correlating them to clinical endpoints specifically chosen for a particular clinical trial.

## 9. Conclusions

The modern development of immunotherapy is marked by significantly higher throughput, cost, and complexity than ever before. Tools are constantly being developed to improve efficiency at each step in the process, and AI may represent a solution for predicting treatment efficacy and minimizing side effects, thereby streamlining the clinical trial portion of novel pharmaceutical testing [42]. When paired with PET imaging, which is already widely used for staging purposes in oncology, AI could allow clinicians to appreciate and quantify the microscopic imaging biomarkers that define a patient’s disease. Even prior to trial onset, researchers could use AI for non-invasive, radiographic tumor phenotyping in order to assess susceptibility to treatment (e.g., evaluating PD-L1 status for patients prior to a trial for anti-PD-L1 monoclonal antibodies) and select patients most likely to achieve clinical benefit. Additionally, while an investigative treatment is being administered, radiographic changes could be used to predict prognosis or signal the insidious development of adverse reactions or atypical response patterns, allowing for increasingly swift reflexes from clinical trial teams. In theory, if a new drug trial were to be associated with poor radiographic phenotypes at first follow-up, it could be terminated at an earlier time point than is currently possible. Further development of AI, combined with high-specificity radiotracers to fully illuminate tumor cell microenvironments, could precipitate a paradigm shift in immunotherapy drug development, dramatically increasing efficiency and decreasing potential morbidity, ultimately facilitating bench-to-bedside transitions for novel therapeutics.

## Figures and Tables

**Table 1:** Data extracted from relevant studies in the current second-look analysis.

Sample Size	Tumor Type	Modality	Task	Secondary Task	Data Collection	Multicenter	Validation Strategy	RQS	Year	PMID	Citation
697	Lung	PET/CT	Tumor phenotype	Prognosis	Retrospective	Yes	Validation set; test set	23	2021	34135101	[13]
399	Lung	PET/CT	Tumor phenotype		Retrospective	No	Validation set	12	2020	31147234	[14]
255	Lung	PET/CT	Tumor phenotype		Retrospective	No	Validation set	15	2021	34976829	[15]
210	Lung	PET/CT	Classification	Prognosis	Retrospective	Yes	Validation set; test set	16	2021	33828255	[16]
194	Lung	PET/CT	Prognosis		Prospective	No	Validation set; test set	24	2019	31807885	[17]
194	Lung	PET/CT	Classification		Retrospective	No	Validation set; test set	24	2020	33937811	[18]
181	Lung	PET	Immune environment	Treatment response	Retrospective	Yes	Test set	15	2020	32929383	[19]
132	Lymphoma	PET	Prognosis		Retrospective	No	Validation set	12	2020	32248365	[20]
103	Lung	PET/CT	Immune environment		Retrospective	No	Validation set	17	2021	34868999	[21]
57	Lung	PET	Prognosis	Treatment response	Retrospective	No	None	9	2020	32380754	[22]
56	Melanoma	PET	Prognosis		Retrospective	Yes	Cross-validation	7	2022	35204479	[23]
52	Melanoma	PET/CT	Prognosis	Classification	Retrospective	No	None	6	2021	33811161	[24]
31	Lung	PET	Tumor phenotype		Retrospective	No	None	6	2020	32894535	[25]
30	Lung	PET	Prognosis		Retrospective	No	Cross-validation	18	2020	32726293	[26]
26	Melanoma	PET/CT	Treatment response		Retrospective	Unknown	None	0	2020	32259852	[27]
